# Supplementary material for: Prevalence and determinants of mother and newborn skin-to-skin contact in Ghana
Source: PLoS One. 2025 Dec 4;20(12):e0337325. doi: 10.1371/journal.pone.0337325 (PMC12677551; doi:10.1371/journal.pone.0337325)
Supplement: Attachment — Submitted filename: Response to Comments_R2.docx [file pone.0337325.s003.docx]

**RESPONSE TO REVIEW COMMENTS (REVISION 2)**

Reviewer #1: I recommend the manuscript is for publication in PLOS One journal. the authors have address most of the comments raised in the previous round of review. However, a few areas still require attention, as noted below.

1.Abstract
The statement in the background could be better expressed as follows: “Despite the well-established role of skin-to-skin contact in reducing neonatal mortality, its implementation varies significantly across geographical regions, particularly in sub-Saharan Africa. Therefore, we estimated the prevalence of mother-newborn skin-to-skin contact at birth and investigated the factors associated with its practice in Ghana.”

Response: We have revised the sentence as suggested to read “Despite the well-established role of skin-to-skin contact in reducing neonatal mortality, its implementation varies significantly across geographical regions, particularly in sub-Saharan Africa. Therefore, we estimated the prevalence of mother-newborn skin-to-skin contact at birth and investigated the factors associated with its practice in Ghana.”

Introduction
Authors were advised to conclude the introduction with a clear statement of the study objectives. They responded by repeating the research title at the end of the introduction. Instead, authors should clearly specify the study objectives rather than restate the title.

Response: We have revised the objectives of the study to read “Therefore, this study aims to (i) estimate the prevalence of mother-newborn SSC in Ghana and (ii) examine the factors associated with the practice of mother-newborn SSC at birth in Ghana”.

Variables
Authors should review paragraph 2, line 6, where “wanted las pregnancy” appears to be a typographical error and make the necessary correction.

Response: We have corrected this to read “last pregnancy”.

Results
Authors should specify the statistical method used to calculate the 95% confidence intervals for the variables in Table 1. This was not explicitly mentioned in the Results section.

Response: We performed a bivariate analysis to estimate the percentages and confidence intervals for mother-newborn skin-to-skin contact across the explanatory variables.
